# Recessions and Health: The Long‐Term Health Consequences of Responses to the Coronavirus*

**DOI:** 10.1111/1475-5890.12230

**Published:** 2020-07-06

**Authors:** James Banks, Heidi Karjalainen, Carol Propper

**Affiliations:** ^1^ Institute for Fiscal Studies University of Manchester; ^2^ Institute for Fiscal Studies; ^3^ Imperial College Business School Institute for Fiscal Studies

**Keywords:** recessions and health, COVID‐19

## Abstract

The lockdown measures that were implemented in the spring of 2020 to stop the spread of COVID‐19 are having a huge impact on economies in the UK and around the world. In addition to the direct impact of COVID‐19 on health, the following recession will have an impact on people's health outcomes. This paper reviews economic literature on the longer‐run health impacts of business‐cycle fluctuations and recessions. Previous studies show that an economic downturn, which affects people through increased unemployment, lower incomes and increased uncertainty, will have significant consequences on people's health outcomes both in the short and longer term. The health effects caused by these adverse macroeconomic conditions will be complex and will differ across generations, regions and socio‐economic groups. Groups that are vulnerable to poor health are likely to be hit hardest even if the crisis hit all individuals equally, and we already see that some groups such as young workers and women are worse hit by the recession than others. Government policies during and after the pandemic will play an important role in determining the eventual health consequences.

## I. Introduction

The current lockdown and social distancing measures brought about by the coronavirus crisis, coupled with the direct effects of the virus on workers and firms, are having a huge impact on economies in the UK and around the world. Existing literature on the health impacts of business‐cycle fluctuations and recessions shows that the resulting economic downturn will have significant consequences on people's health outcomes in the short and longer term. This paper discusses some of the mechanisms through which shocks to macroeconomic conditions may affect health.

An economic downturn has a number of effects on people's lives through increased unemployment, decreased employment, reductions in income and wealth, and increased uncertainty about future jobs and income. The health effects caused by these adverse macroeconomic conditions will be complex and will differ across generations, regions and socio‐economic groups. Groups that are vulnerable to poor health are likely to be hit hardest even if the crisis hit all individuals equally, but evidence is already emerging that the economic repercussions of the crisis are falling disproportionately on young workers, low‐income families and women,^1^ so this will also need to be taken into account. Policies that the government has put in place, as well as the subsequent policies that will be implemented both for the ‘exit strategy’ and for post‐pandemic economic recovery, will play an important role in determining the eventual health consequences.

This paper discusses what we know about the long‐run health effects of business‐cycle fluctuations and recessions, but there are additional aspects of the coronavirus crisis that are unique to the current situation that stem from this being a pandemic. Most obviously, the crisis has an impact on the availability of healthcare. Initially, all non‐urgent elective treatments were postponed, and the need for social distancing is making resumption of normal levels of service difficult. The aftermaths of this additional strain on the NHS on future healthcare are addressed by Propper, Stoye and Zaranko (2020, this issue).

Declines in economic activity may lower mortality rates during the recession itself, due to reductions in industrial accidents, traffic accidents and pollution‐related deaths.^2^ But recessions and economic downturns have been shown to have negative effects on subsequent longer‐run mortality. And looking beyond mortality, morbidity has been shown to increase during recessions, and this is particularly true when long‐run dynamics, spillovers and spatial effects are taken into account. Janke et al. (2020) estimate a model of the impact of economic shocks on morbidity for Britain that allows for different responses by local area, for persistence in the effect of past shocks and for feedback from national changes in levels of morbidity to the local level. They find that employment changes during and after the 2008 financial crisis had a strong adverse effect on chronic health for five broad types of health conditions, with the strongest effects being for mental health conditions.^3^

Quantitatively, Janke et al. estimate that a 1 per cent fall in employment leads to a 2 per cent increase in the prevalence of chronic illness. To put this in context, if employment were to fall by the same amount as it fell in the 12 months after the 2008 crisis, around 900,000 more people of working age would be predicted to suffer from a chronic health condition. Only about half this effect will be immediate: the full effect will not be felt for two years. The shock to employment from the coronavirus pandemic is likely to be much larger than this and so we may expect a larger rise in poor health. The Janke et al. analysis looks at the prevalence of long‐standing health conditions but does not examine the intensity or the duration of the condition within an individual's life course. It is quite possible that health status, outcomes and levels of functioning may well continue to deteriorate over the longer run even once the prevalence of chronic long‐standing conditions has plateaued.

## II. Looking beyond average effects

The Janke et al. (2020) estimate is of an average effect, across all ages, areas and income groups. In any recession, many groups will experience worse‐than‐average changes in economic fortunes. It is likely that the groups that suffer the biggest economic losses from this crisis are also those who were more vulnerable to begin with – for example, people with lower incomes are less likely to be able to work from home or have accumulated liquid savings to tide them over. In addition, even the same change in economic fortunes may have different health consequences for different groups. Research from many fields has shown that certain individuals are more resilient to shocks than others and that there are particular periods in life where shocks are most critical for long‐term outcomes. Groups of particular concern are families with young children or where mothers are pregnant, and low‐income or low‐socio‐economic‐status individuals of all ages where health vulnerabilities and mental health problems are already prevalent.

Economic shocks and downturns have been shown to be important during pregnancy and early childhood. At this point of the life cycle, physical health and cognitive trajectories are being set and this has long‐reaching consequences for later‐life economic outcomes and later‐life health. Poverty rates increase in a recession. This may feed through to negative *in utero* and early childhood risk factors. Van den Berg, Lindeboom and Portrait (2006) examine the Dutch population born between 1812 and 1912 and find that the state of the business cycle at birth affects mortality: being born in a recession reduces lifespan by about 5 per cent. This illustrates the stark and lifelong effects that early childhood conditions have on health, not just because of the biological pathways, but also as a result of the effects of *in utero* and early childhood health conditions on other economic outcomes and family behaviours.

Currie (2009) documents the extensive evidence on the links between parental circumstances and child health, as well as the subsequent link between a child's early‐life health and their eventual educational and labour market outcomes. It is well documented that poor nutrition in early childhood, as well as *in utero*, will have a long‐lasting impact on individuals, and there are many other examples where vulnerabilities and shocks in early life have long‐term consequences. Hence the interaction of children's age and their family's vulnerability will be a key point when thinking about policies to mitigate the long‐run health and economic effects of the crisis.

Those with pre‐existing poor mental health will also be particularly vulnerable. The adverse impact of recessions on mental health and mortality from suicide is clearly documented across a number of studies.^4^ Prevalence of mental health conditions in the UK has been rising even before the coronavirus crisis, and particularly so for the young, making this a particularly important outcome to consider. Estimates drawn from Janke et al. (2020) suggest that if the economic downturn were similar to that after the 2008 financial crisis, the number of people of working age suffering from poor mental health would rise by half a million. Poor mental health and depression are also a risk factor for a number of physical health conditions,^5^ and interventions targeting improved mental health can reduce future hospitalisation.^6^ Thus the impact of the economic downturn on mental health will also affect overall long‐run health and mortality through this channel.

To add to this, social distancing in itself is likely to have complex and nuanced effects on individuals’ social isolation and mental health, which will in turn feed through into physical health and mortality.^7^ These social distancing effects of the coronavirus crisis will be additional to any mental health effects that might be predicted when extrapolating from analysis of previous downturns and are hard to predict, particularly when taking into account that these experiences are shared by the whole population and can simultaneously cause a sense of ‘togetherness’ that may ameliorate some of the negative effects. Nevertheless, the early emerging evidence is that the mental health consequences of the pandemic in the UK have been substantial.^8^

Reduced economic activity as a result of a recession may also have some positive health impacts over and above any reduced mortality. Some unhealthy behaviours such as drinking, smoking and unhealthy eating have been shown to fall, on average, when there are negative income shocks,^9^ although how these react during a lockdown‐induced recession will be important to document. Reports already show reduced levels of air pollution in the industrial areas of China and Italy as well as London, and there is a clear link between mortality from certain cardiovascular and respiratory causes and air pollution.^10^ Moreover, the rate of accidents in traffic or at work will be lower given fewer cars on the roads and reduced industrial activity. And in addition to the short‐term effects of the social isolation in slowing down the spread of the coronavirus, Adda (2016) finds that economic downturns slow down the spread of viral disease as interregional travel and trade are reduced. Thus even after the social distancing measures have been lifted, we can expect the rate of viral transmission to be lower in an economic downturn than it would have been otherwise.

## III. Potential structural changes will have a long‐lasting impact

In the long run, the current economic downturn and the social distancing measures that accompany it will hit some industries more than others, and consequently some regions more than others. Janke et al. (2020) find heterogeneous morbidity responses to economic shocks across local areas. The hardest‐hit areas are those that are the most deprived, have older populations and have older industrial structures, which are precisely the kind of areas least able to withstand negative shocks.

The coronavirus crisis has the potential to create long‐lasting structural changes in the UK economy. In terms of industries, travel and tourism, as well as hospitality and any non‐essential retail trade, have come to a complete halt since the lockdown was imposed. Areas of the UK that are disproportionately reliant on one of these sectors may see a severe collapse of activity in the local economy if recovery after the crisis is not fast enough. American studies of areas that were most exposed to structural shocks and industrial decline due to the rise of trade between the US and China have shown long‐lasting and severe effects, particularly for males.^11^ These shocks have fed through to mortality outcomes.^12^ This is a specific example of how changes in economic fortunes can lead to ‘deaths of despair’, the term coined by Case and Deaton (2015 and 2017), who document strikingly large rises in mortality associated with suicide and alcohol and drug abuse amongst low‐educated white American males. The health effects of wholesale structural change, where industries are eradicated in a local area, are severe and persistent as the economic opportunities of people in these areas disappear either permanently or at least for decades.

## IV. Government policy can play a role in alleviating adverse health consequences

In facing this economic downturn, government intervention will play a key role in determining the eventual health effects of the resulting recession. The government will need to decide where resources are best used. Importantly, in recent years, the UK welfare system has evolved to protect incomes through the extensive use of in‐work benefits. Whilst this proved to be a positive during the 2008 financial crisis when incomes and wages fell but employment held up,^13^ continued erosion of the value of out‐of‐work benefits relative to average wages leaves the system far less capable to deal with mass unemployment shocks than it was.

The Coronavirus Job Retention Scheme (CJRS) has a specific purpose of preserving jobs as well as incomes and is desirable in this respect.^14^ Obviously, within the lockdown period, most of those who do lose their jobs will find it very difficult to find new work, but the CJRS should help to ensure that once the social distancing measures are lifted, more have retained their jobs and can return to work. In the absence of measures that protect employment, job separation and long spells of unemployment can lead to a loss of human capital either through depreciation in skills or a loss of firm‐specific human capital that has accumulated through matching in the labour market.^15^ This will have long‐run consequences for individuals’ lifetime earnings as well as affecting their financial security, sense of purpose and mental health. Thus we would also expect knock‐on adverse effects on morbidity and mortality. Therefore, protecting human capital and minimising unemployment consequences beyond the duration of the social distancing measures are particularly important for both the economic and health consequences of the recovery. Rebuilding lost human capital may well be much more complex than replacing the physical or financial capital that has been wiped out in the large recessions experienced recently.

The example of the CJRS shows that even the policies that are not directly aimed at improving health outcomes can help alleviate the eventual health consequences of the economic downturn. Thus it is important that any government action is taken with consideration for the long‐term health outcomes they may affect. Different policies will help certain groups more than others, and affect their long‐term health in differing ways. For example, it is clear that the long‐run returns from supporting vulnerable mothers and children through this crisis will have far‐reaching consequences for the outcomes of the children. But the current policies aimed at supporting furloughed workers may not do enough to reach these families. Thus any policy should come with an assessment of who it will benefit most and how the health effects may differ by cohort, prior socio‐economic status and region. This will be particularly important when the government designs its policies for the removal of the social distancing measures and any subsequent reimposition of them, and then again as additional government economic support measures are phased out.

## Footnotes

1 Joyce and Xu, 2020.

2 Ruhm, 2000.

3 Not all recessions are the same of course, so the extent to which studies of past recessions can be generalised to the upcoming 2020 COVID recession should be borne in mind. Cribb, Hood and Joyce (2017) show that the 2007–08 recession was different from the preceding two recessions in that the incomes of the poor were relatively well protected, and one would expect this to track through to its subsequent health consequences. Indeed, Coveney et al. (2020) show that the 2008 financial crisis and subsequent recession did not widen health inequalities in Europe, as might have been predicted from previous recessions.

4 World Health Organization, 2011.

5 Kivimäki et al., 2018.

6 Gruber et al., 2019.

7 See, for example, Shankar et al. (2011) or Steptoe et al. (2013).

8 See Banks and Xu (2020), for example.

9 Ruhm, 2000; Adda, van Gaudecker and Banks, 2009; Griffith, O'Connell and Smith, 2016.

10 See, for example, Janke, Propper and Henderson (2009).

11 Autor, Dorn and Hanson, 2016 and 2019.

12 Pierce and Schott, 2020.

13 See Cribb, Hood and Joyce (2017).

14 See Costa Dias et al. (2020, this issue) for further commentary on the CJRS.

15 Fujita, Moscarini and Postel‐Vinay (2020) further discuss the importance of preserving workers’ attachment to their current employer in the context of the coronavirus crisis.
